# Diagnosis of Sub-Acute Ovaritis

**Published:** 1873-12

**Authors:** Edward John Tilt


ON THE DIAGNOSIS OF SUBACUTE OVARITIS.—*By Edward John Tilt, M.D.*—The author suggested that the undervaluing of the part played by subacute ovaritis as a source of disease in women partly depended on the lamentable facility with which many practitioners, wherever there was pain in the ovarian region, inferred the existence of ovaritis, partly on account of the real difficulties of diagnosis, of which he gave some remarkable instances. He intimated that another reason might, however, be found in the difficulty of making examinations in young unmarried women. He had found that the most frequent sexual diseases during this period of life (between fifteen and twenty-five) were subacute ovaritis and inflammation of the neck of the womb. When the disorders of menstruation resisted good hygienic and medical treatment, he believed they were generally due to subacute ovaritis and cervicitis. The symptoms of cervicitis he described to be the habitual painless passing of a moderate amount of muco-purulent vaginal discharge with habitual pain in the back; those of subacute ovaritis were constant dull pain deep in the ovarian region, much increased by firm pressure and extending to the thigh and leg, mammary symptoms, disturbed menstruation, and hysterical phenomena. The positive sign of subacute ovaritis was the finding of an ovid, smooth, or slightly indented lump beside the womb or in Douglas's pouch, pressure upon which, caused by the practitioner's finger, or during coitus, caused an overpowering and sickening sensation of pain and debility. It might be necessary to confirm this diagnosis by a rectal or recto-vaginal examination. The author expressly stated that it would sometimes occur to a practitioner making a first vaginal examination, that instead of finding any ovarian disease, as he expected, he would detect cervical disease; and in other cases subacute ovaritis would be found when the symptoms would lead him to expect cervical inflammation. He concluded by describing the line of conduct to be adopted by the surgeon for the management of each of the three classes, and sketched the treatment most likely to cure them.

Dr. Barnes thought that many ovarian symptoms had their origin in uterine displacement.

Dr. Wynn Williams concurred in Dr. Barnes's opinion. He had rarely met with a case of ovaritis without some uterine complication, unless caused by violence. He believed the uterine mischief preceded the ovarian, and his experience taught him that on the removal of the uterine ailment the ovarian soon subsided. The same remarks applied to the ovary as to the testicle. Orchitis was seldom met with without previous inflammation of the urethra. He had frequently relieved ovarian symptoms by introducing a stem into the uterus, and he had under his care several unmarried females wearing stems and shields, who could not be persuaded to have them removed, dreading a return of their sufferings.

Dr. Haywood Smith believed the best way of using the double touch was to use the forefinger of the right hand for the vagina, and the middle finger of the same hand for the rectum, the left hand was thus left free to make pressure on the hypogastric region. With regard to what had been stated as to ovaritis being associated with or consequent upon flexions or other morbid states of the uterus, he must say that he frequently found patients suffering from ovaritis in various stages unconnected at all with any flexions or misplacements of the uterus, or with any metritis.

Dr. J. J. Phillips said that his experience was, that so-called subacute ovaritis was a very frequent cause of dysmenorrhea and he believed that this ovaritis frequently exists independently of any uterine flexion or peculiar conformation of the cervix uteri. It was very common to find a swollen hyperemic, probably inflamed, ovary as the only evidence of disease in cases of dysmenorrhea. The fact stated by Dr. Barnes, however, could not be controverted, that in a large class of cases dysmenorrhea was due to uterine flexion, or some uterine abnormality, and by appropriate treatment not only was the pain relieved, but also the congested state of the uterine appendages. A suitable selection of cases was, however, important, for some of the most troublesome cases of ovaritis and peri-oophoritis had their origin in mechanical interference with the neck of the uterus by incision or dilatation.

Dr. Gervis remarked that the truth might be between the two views which had been expressed, and that while primary subovaritis was probably rare, yet it might certainly continue to exist as a result of various uterine affections, such as chronic endometritis and mechanical dysmenorrhea, after these affections had been cured.

Dr. Tilt, in reply, stated he had always taught that mechanical obstructions to the menstrual flow and uterine mal-positions required especial treatment, but he intentionally omitted to treat of them in the present instance, because they were not of very frequent occurrence between the ages of fifteen and twenty-five, whereas he had come to the conclusion that subovaritis and cervicitis were then common, and were often treated as diseases of menstruation; he did not think that the analogy between the testicle and the ovary should be pushed too far, because the testicle was not subject to any monthly process similar to ovulation.

